# Functionalizing PAN Carbon Nanofibers Using Ti_3_C_2_T_x_ MXene for Improved Thermal and Electrochemical Behavior

**DOI:** 10.1002/marc.202500429

**Published:** 2025-08-20

**Authors:** Fatemeh Mokhtari, Thomas Groetsch, Pejman Heidarian, Maxime Maghe, Russell J. Varley

**Affiliations:** ^1^ Department of Materials Engineering KU Leuven Leuven Belgium; ^2^ Carbon Nexus at the Institute for Frontier Materials Deakin University Geelong Victoria Australia; ^3^ School of Engineering RMIT University Melbourne Victoria Australia

**Keywords:** carbon nanofiber, electrospinning, energy storage, MXene, polyacrylonitrile

## Abstract

Electrospinning and dip coating are well‐established and highly effective methods to incorporate 2D Ti_3_C_2_T_x_ MXene flakes into nanofibers. The synergies exhibited between MXene and carbonized nanofiber (CNF) networks significantly enhance nanofiber performance in energy and environmental applications, demanding further investigation into MXene incorporation strategies. This study systematically evaluates different strategies for incorporating MXene flakes into electrospun polyacrylonitrile (PAN) fibers under consistent processing conditions, followed by an investigation into the thermal and electrochemical behavior of the resulting CNF mats. The first strategy incorporates MXene into the electrospinning solution, creating a uniform dispersion within the resultant nanofiber. In the second approach, MXene is deposited onto the mat via dip coating, while the third approach combines both methods, searching for synergies through enhanced distribution and surface functionalization. All mats were thermally stabilized at 260°C and subsequently carbonized at 900°C. Thermogravimetric analysis was employed to evaluate the thermal stability of the mats throughout the pre and post‐carbonization processes. The MXene coating approach reduced mat shrinkage, leading to an increased carbon yield after carbonization, while incorporating MXene directly into the nanofiber solution provided the highest CNF flexibility. The third approach achieved the highest MXene loading, enhancing electrical conductivity and pseudocapacitive performance.

## Graphical abstract

A study of dispersion and coating strategies of Ti_3_C_2_T_x_ MXene flakes into electrospun polyacrylonitrile fibers under consistent processing conditions, after oxidation and carbonization, showed that the application method profoundly impacted properties. Coating the fiber reduced mat shrinkage and increased carbon yield, direct incorporation provided the highest flexibility, while a combined approach achieved the greatest enhancement in electrical conductivity and pseudocapacitive performance.

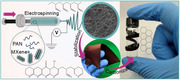

## 1 Introduction

Carbon fiber is the material of choice for lightweight, high‐performance composite applications due to its exceptional tensile strength, low density, and superior thermal and chemical stability [1, 2]. This exceptional performance has led to the integration of 2D with carbon fibers to create multi‐functional composites for enhanced electrochemical energy storage (EES) systems and devices. Amongst these EES technologies, supercapacitors (SC) stand out as prime candidates for energy storage due to their superior power density, rapid charge‐discharge capabilities, excellent cycling stability, environmental friendliness, and cost‐effectiveness [3]. SCs are widely used in applications such as portable electronic devices (smartphones, laptops, mobile power supplies), electric vehicles, and off‐grid systems [4].

MXenes are a family of 2D transition metal carbide/nitrides (equation: M*_n_*_+1_X*_n_* T*_x_*, where M = early transition metal, X = carbon and/or nitrogen, *n* = 1–4, and T*_x_* = –F, –O, −OH) which have attracted significant attention as promising electrodes in supercapacitors due to their excellent electrical conductivity and fast ion transfer capability [4, 5]. Supercapacitor electrodes require high conductivity to enable rapid charge/discharge cycles and efficient ion transport [6]. MXene, with its high specific surface area and layered structure, facilitates the diffusion and binding of ions more efficiently, resulting in enhanced power density [7]. Depending on the synthesis techniques, MXene surface termination groups (─F, ─OH, ─Cl, or ─O) are formed, affecting its hydrophilic properties, which play a key role in the electrochemical performance of MXene‐base electrodes in supercapacitors [8, 9].

It has recently been shown that embedding MXenes within carbon nanofiber composite electrodes limits redox reactivity at the electrode/electrolyte interface, reducing capacitance efficiency [10]. To overcome this, a conductive coating is applied to the electrode surface using electrodeposition [11], hydrothermal deposition [12], chemical grafting [13], or dip‐coating [14]. Among these, dip‐coating in particular is a simple and cost‐effective technique to produce a highly redox‐active electrode, as demonstrated recently using an MXene (Ti_3_C_2_T_x_) colloidal solution [10]. While the dip‐coating technique is a promising approach, significant challenges remain as it tends to compromise the flexibility of the electrodes, causing the active material to flake off and reduce conductivity [15]. Additionally, increasing the electrode mass during the dip‐coating process can lead to restacking of the MXene flakes, further obstructing ion diffusion and degradation of the electrochemical performance [15].

The inadequate strength of MXene films arising from the weak interactions between flakes can be improved using a polymer binder. However, solution‐based preparation methods often cause significant aggregation of MXene flakes, leading to defects and unevenness in the films, which in turn reduces their performance. To avoid MXene flake stacking, electrospinning presents a promising solution as it effectively aligns the 2D MXene flakes into a 1D configuration along the radial direction of the nanofibers [16].

Electrospinning (ES) is a straightforward and low‐cost technique for producing nanoscale fibers at scale [9]. It is widely employed to incorporate nanomaterials into fibers while controlling fiber diameter and orientation [17]. Electrospun mats fabricated through ES offer advantages such as high surface area and high porosity [18]. Carbonizing polymer fibers after ES enables the grafting of nano compounds, such as carbon nanotubes (CNTs), graphene, and metal–organic frameworks (MOFs), onto carbon nanofibers. These composites can serve as binder‐free electrodes for energy storage, providing excellent conductivity, low weight, good chemical stability, and a self‐standing structure [14]. In a recent study, a hydrothermal method was applied following the carbonization process to convert a portion of the embedded MXene within CNFs into sodium titanate. This approach aimed to enhance conductivity (Rs ≤ 4 Ω), prevent self‐agglomeration, and expose a greater number of electrochemically active sites due to the resulting spiky morphology [19].

Carbon nanofibers derived from carbonized electrospun polymer fibers demonstrate outstanding physicochemical and textural properties, making them a promising material for enhancing electrochemical double‐layer capacitance [20]. The combination of MXene flakes and PAN, followed by electrospinning, has been widely used to fabricate multifunctional nanofibers in various studies [16, 21]. Additionally, hollow Ti_3_C_2_T_x_ MXene/carbon nanofibers produced via electrospinning can be used as anode materials for lithium‐ion batteries (LIBs) [22].

Recent studies have explored both the dip‐coating of MXene onto fibers [4, 23] and the embedding of MXene in nanofibers [11, 24]. These investigations primarily focused on the use of carbon nanofibers in applications such as electromagnetic interference (EMI) shielding and supercapacitor electrodes, highlighting MXene pseudocapacitance, metallic conductivity, and rapid faradaic reactions [25]. However, a key issue in comparing performance lies in the variations in MXene quality, size, concentration, PAN precursor composition, and the conditions of the stabilization and carbonization processes, such as residence time and temperature. These inconsistencies across studies complicate direct comparison, and in particular, research has shown that the size of MXene flakes plays a significant role in the electrochemical performance of PAN‐based flexible supercapacitor electrodes [26]. Therefore, it is crucial to maintain consistent MXene parameters when comparing the performance of electrodes fabricated using different methods. However, studies on the formation of carbon nanofibers, their electrochemical performance, and thermal properties, particularly in relation to the various methods of incorporating MXene into carbon nanofibers, have yet to be reported.

This study focuses on evaluating MXene incorporation methods under controlled conditions for their effects on thermal and electrochemical performance. Ti_3_C_2_T_x_ MXenes were successfully synthesized through a minimally intensive layer delamination (MILD) process. The MXene was incorporated into electrospun PAN nanofibers using methods including in situ embedding of MXene into PAN nanofibers (PM), ex situ dip coating of MXene onto nanofibers (P+M), and a combination of both approaches (PM+M), which were compared with neat PAN nanofibers (P). All electrospun mats were heat‐treated under controlled stabilization and carbonization (up to 900°C) processes using pilot‐scale carbon fiber production equipment. By maintaining constant process variables, the thermal behavior of electrospun mats was investigated at each stage of the manufacturing process, from precursor to carbonized material. Additionally, the resistance and electrochemical performance of the carbonized mats were studied.

## 2 Result and Discussion

### 2.1 Morphology and Structure Characterization of Nanofibers

**FIGURE 1:**
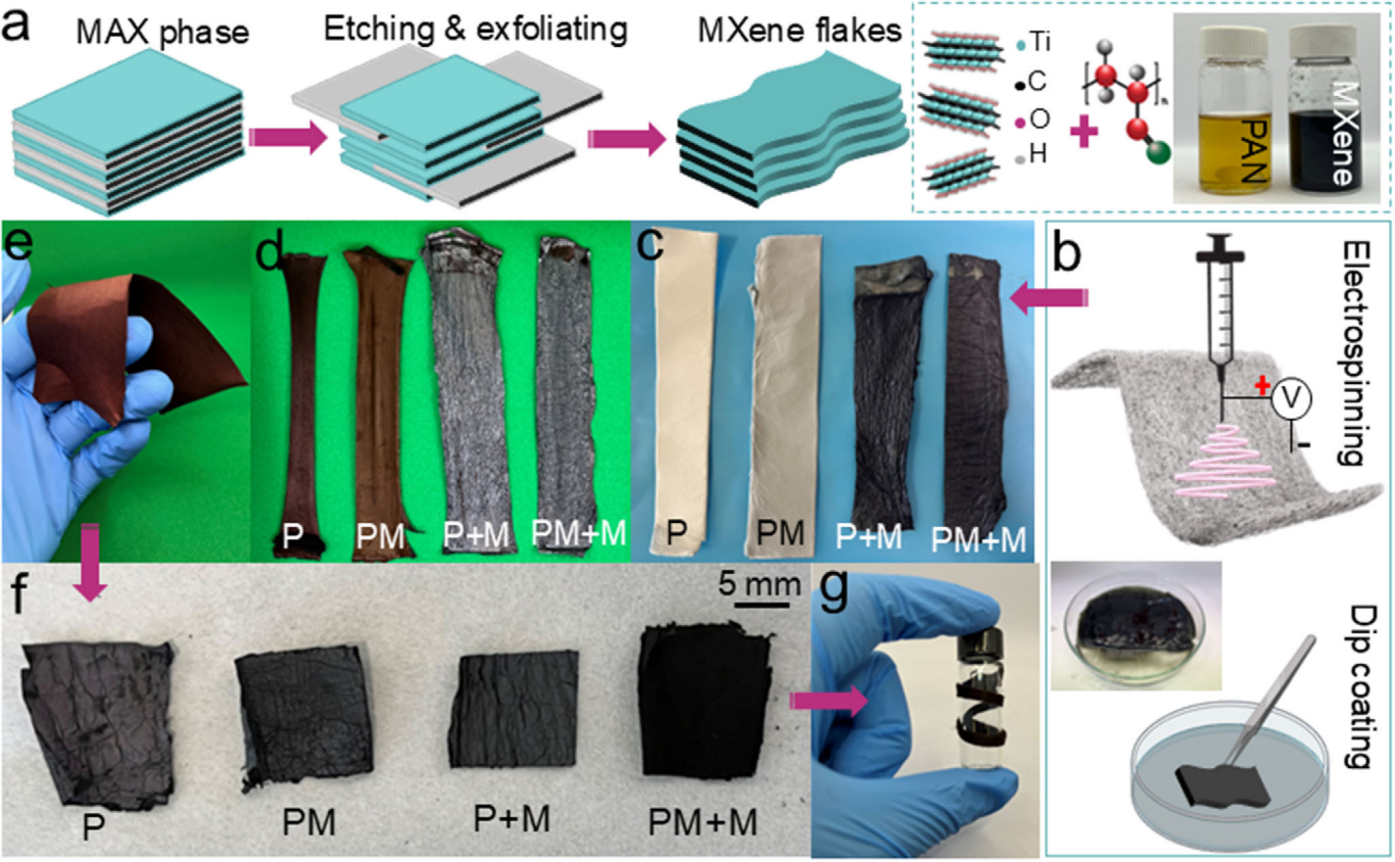
(a) Schematic of synthesis of Ti_3_C_2_T_x_ MXene, delaminated MXene flakes were dispersed in DMF, then blended in PAN to prepare the PAN/MXene solutions, (b) nanofiber mats were fabricated via electrospinning followed by dip‐coating in MXene solution of ethanol/water, Photographs of the (c) electrospun fiber mats: neat PAN (P), MXene‐incorporated (PM) and MXene‐coated (P+M, PM+M), (d) stabilized mats, (e) demonstrating the flexibility of stabilized mats (f) carbonized mats, and (g) the flexibility of the PM mat was demonstrated by wrapping it around a glass vial.

Figure 1 shows the overall procedure for the fabrication of the MXene modified CNF mats. MXene (Ti_3_C_2_T_x_) flakes were synthesized by selectively removing the aluminum layer from the precursor MAX phase (Ti_3_AlC_2_), affording OH, –O, and –F surface groups. These improve wettability and promote ion transport, enhancing redox performance [27]. MXene flakes were then uniformly dispersed in a PAN and dimethylformamide (DMF) solution, forming a homogeneous blend through magnetic stirring (Figure 1a). After electrospinning, some nanofiber mats were dip‐coated in a MXene solution (Figure 1b) and displayed a gradual color change from white for neat PAN (P), to gray for mats containing 1 wt.% Ti_3_C_2_T_x_ MXene (PM), and black for the MXene‐coated mats (P+M, PM+M) (Figure 1c). This visual transition reflects increasing MXene content and surface loading, which aligns with structural changes observed post‐carbonization

The nanofiber mats were stabilized at 260°C for one hour under tension, became darker, either brown or black, and flexible (Figures 1d, e). The purpose of stabilization is to transform the linear structure of PAN into an intractable ladder‐like unsaturated cyclic polymer, yielding high levels of aromatization, facilitating carbonization at elevated temperatures [28]. The significantly higher surface to volume ratio of nanofibers, compared to conventional carbon fibers with micrometer diameters size, required extensive optimization of the processing parameters. In this study, the extent of cyclization reaction (EOR) was determined to be 60% (Equation 1) to allow sufficient stabilization for effective carbonization while not making the fiber too brittle.

Stabilization produces the greatest mass loss in the neat PAN mat (P) (13.4%) and the least in the MXene‐coated mat (P+M) (6%), resulting in a large disparity in the shrinkage of the mat. The reduced shrinkage of the MXene‐coated mats can be attributed to the high thermal stability of MXene flakes and has a profound impact on the structural integrity of the mat (Figure 1d). Among the MXene‐containing mats, the PM mat showed the highest mass loss (8.6%), despite having the greatest flexibility, due to the superior heat conduction properties of MXene, enhancing heat transfer within the fiber [29]. After 9 min of carbonization in a nitrogen atmosphere, black free‐standing mats were obtained (Figure 1f). Despite this, the PM mat exhibited the highest flexibility, being able to be easily wrapped around a glass vial, forming free‐standing electrodes suitable for flexible devices (Figure 1g).

**FIGURE 2:**
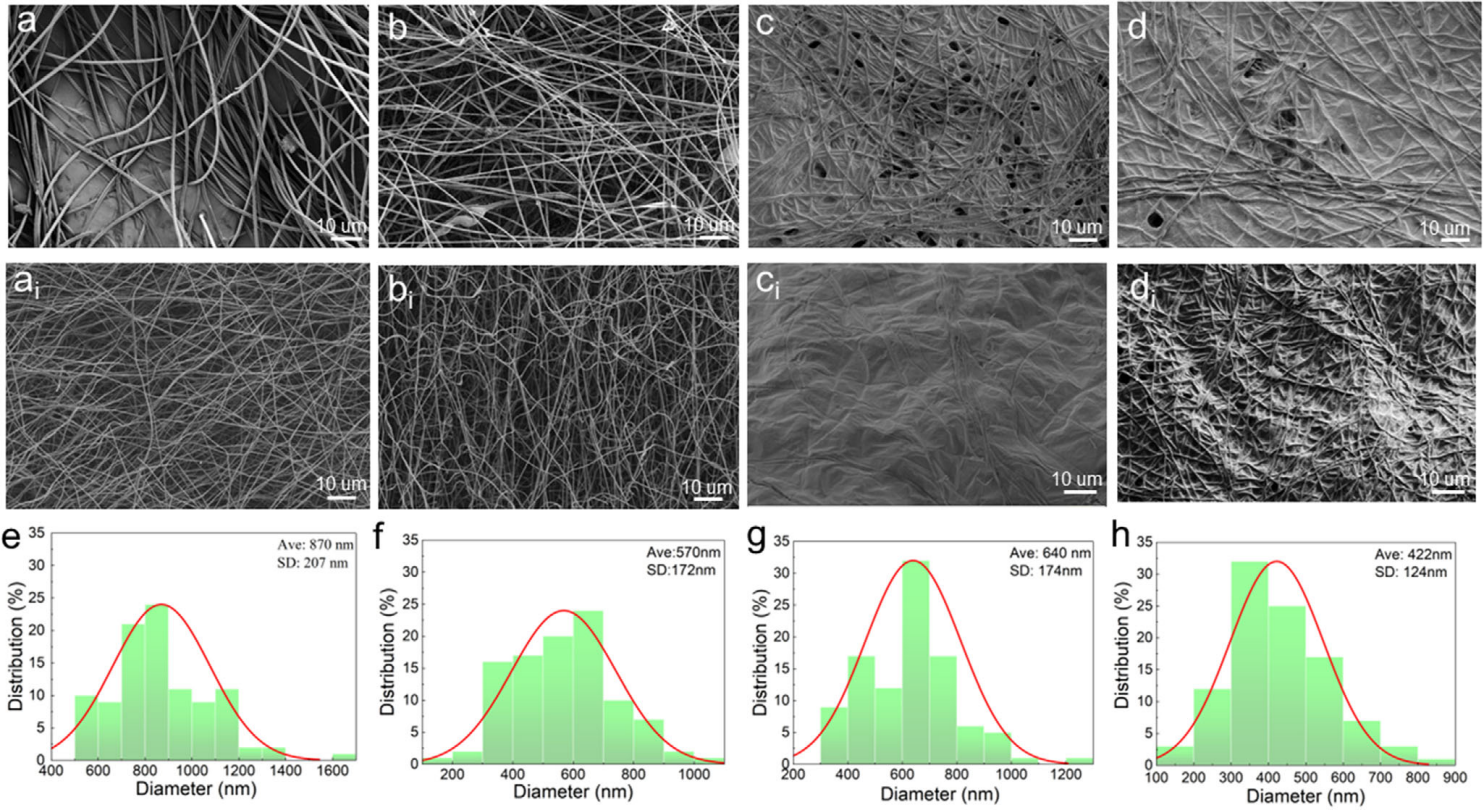
SEM images of electrospun nanofibers: (a–d) before and (a_i_‐d_i_) after carbonization for (a) P, (b) PM, (c) P+M, (d) PM+M nanofibers, fiber diameter distribution for PAN nonaofiber (P): (e) before, and (f) after carbonization, MXene embedded in PAN nanofiber (PM): (g) before and (h) after carbonization.

SEM analysis was performed to examine the microstructure and surface morphology of electrospun PAN‐based composite nanofibers (Figure 2). As the concentration of PAN (12 wt.%) is well above the threshold for uniform fiber formation (≥5 wt.%), uniform fibers without beading and branching were formed [28]. The average fiber diameters for PAN and PAN/MXene were 870 nm and 640 nm, respectively, exhibiting a ∼26% reduction in fiber diameter after the addition of MXene (Figure 2a,b). The increased conductivity due to MXene addition enhanced the electrostatic field during spinning, leading to finer and more uniformly distributed fibers [30]. Importantly, carbonization at 900°C did not significantly change the morphology of these nanofibers despite a 35% reduction in fiber diameter (Figure 2e–h). All nanofiber mats retained their fibrous structure, with no collapse or aggregation, providing ideal conditions for forming a 3D conductive network.

**FIGURE 3:**
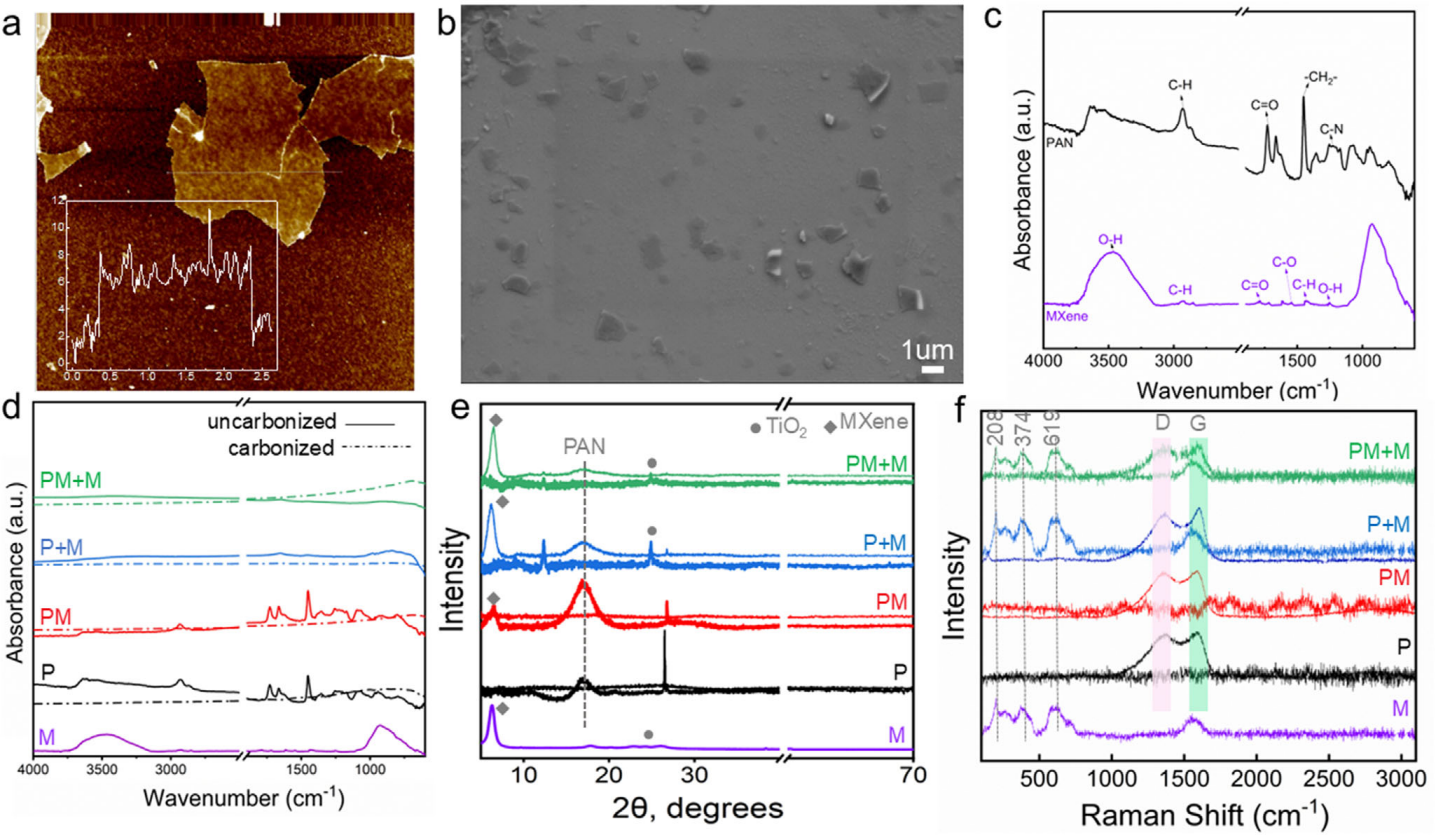
(a) AFM of Ti_3_C_2_T_x_ MXene on Si substrate before sonication, (b) SEM of MXene flakes after prob sonication in DMF, (c) FTIR spectra of MXene and nanofiber before carbonization, (d) FTIR of all CNF before and after carbonization, (e) XRD pattern and (f) Raman spectra of CNF with integrated MXene, and (g) magnified peaks for D and G bond for CNFs.

The synthesized MXene exhibited a lateral size of less than 1 µm and a monolayer flake thickness of approximately 6 nm (Figure 3a). However, following solvent exchange and probe sonication, the flake size was reduced to below 500 nm (Figure 3b; Figure S1). The FTIR analysis in Figures 3c,d revealed distinct characteristic peaks corresponding to specific functional groups within the polymer chain. The FTIR spectrum of the pure Ti_3_C_2_T_x_ MXene film shows two main regions. In the 4000–1400 cm^−1^ range, the peaks associated with water include O–H stretching at 3600–3200 cm^−1^ and −OH surface terminations at 1500–1300 cm^−1^. Other peaks attributed to carbon bond vibrations include C–H stretching at 3000–2800 cm^−1^, C═O stretching at 1750–1700 cm^−1^, C–O stretching at 1700–1550 cm^−1^, and C–H bending at 1500–1400 cm^−1^ [31]. In PAN nanofibers, typical absorption peaks appear at 2920 cm^−1^ (C–H), 2240 cm^−1^ (C–N), 1734 cm^−1^ (C═O), 1450 cm^−1^ (─CH_2_‐), and 1240 cm^−1^ (C–N) (Figure 3c) [14]. Following carbonization, the vibrational peaks of ─CH_2_, ─CH, ─CN, and C–C vanished, confirming completion of the carbonization process and the decomposition of organic bonds to form carbon (Figure 3d).

The XRD peaks for PAN nanofibers appear at diffraction angles (2θ) of approximately 16.9° and 26.5° [32]. The initial peak at 16.9° signifies the PAN polymer crystalline structure, corresponding to the (110) crystallographic plane, whereas the second peak at 26.5° matches the (111) crystallographic plane. A wide peak near 23° is attributed to carbon, while peaks at 27.4°, 36.0°, and 54.1° correspond with TiO_2_ phases, indicating the formation of TiO_2_ on the Ti_3_C_2_T_x_ MXene surface during the stabilization and carbonization process [26]. At elevated temperatures and exposed to the ambient air and humidity conditions, Ti_3_C_2_T_x_ MXene flakes react with oxygen (O_2_), resulting in the formation of titanium dioxide (TiO_2_), amorphous carbon solids, and various gaseous byproducts, including H_2_, CO, CO_2_, and HF [33, 34]. Notably, the intensity of these peaks was sharper in the mats coated with MXene, likely due to the exposure of MXene to ambient conditions (Figure 3e).

Figure 3f presents the Raman spectra of the electrospun mats, which exhibit peaks at 208, 374, and 619 cm^−1^, characteristic of titanium carbide (Ti_3_C_2_) [35]. Two relatively weak peaks at 394 and 635 cm^−1^ were observed in the MXene‐coated mat, corresponding to Ti–C bonds in MXene [4, 21]. These peaks are associated with the B1g(1) and B1g(2) vibrational Ti─C modes [36]. The typical Raman modes for the D and G bands, located at 1350 and 1590 cm^−1^, are observed for carbon (Figure 3g). The D peak represents the in‐plane vibration of sp^3^‐bonded carbon atoms, indicating defects in the graphite lattice and an amorphous structure, while the G peak is assigned to the sp^2^‐bonded corresponding to organized and highly oriented graphite crystals [37]. The value of *I*_D_/*I*_G_ decreased as a result of Ti_3_C_2_T*_x_* addition, signifying that MXene may act as the nucleus to induce and promote the CNF crystallization, further confirming its interaction with the PAN matrix [38]. The lower ratio for P+M is thought to be caused by the well‐constructed graphitic structure of MXene on the surface of the mat [39].

**FIGURE 4:**
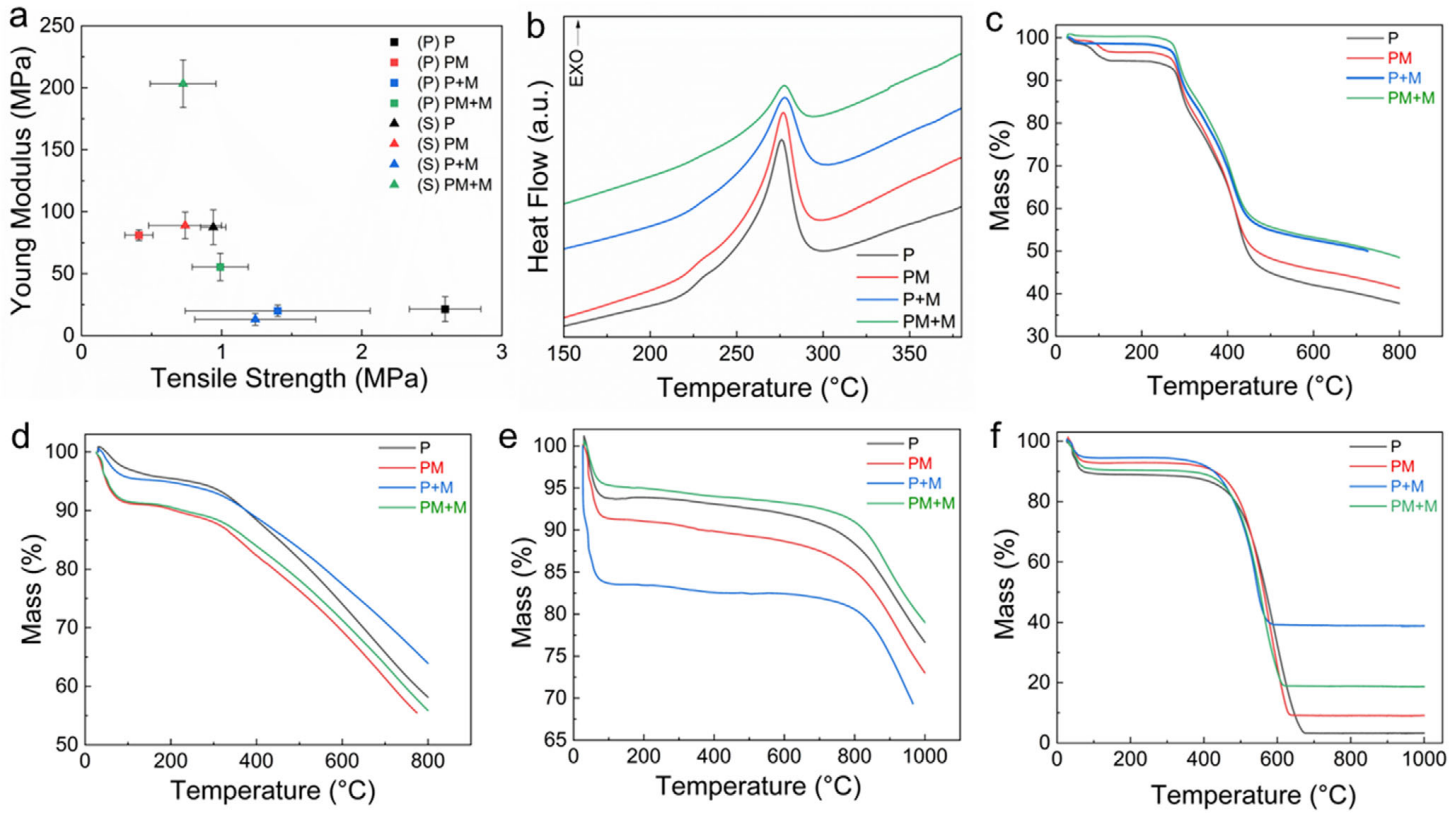
(a) mechanical properties of precursor (P) and stabilized (S) electrospun mats, (b) DSC heating curve of precursor PAN nanofirbers with MXene in nitrogen, TGA results for mats throughout conversion process (c) precursor nanofibers in nitrogen, (d) stabilized nanofiber mats in nitrogen, (e) carbonized mat in nitrogen, and (f) carbonized mats in air.

The tensile strength and Young's modulus of precursor and stabilized electrospun mats are presented in Figure 4a. The tensile strength of the mats decreases after stabilization, a trend typically observed in PAN‐based materials undergoing the well‐established conversion reactions prior to carbonization [40]. Incorporation of MXene into the electrospinning solution enhances the Young's modulus compared to pure PAN. This effect is attributed to MXene's role in preserving a more robust microstructure throughout the stabilization process. The increase in Young's modulus during stabilization for all mats is consistent with trends commonly reported for conventional PAN processing [41]. However, this trend differs for the coated mat (P+M).

The DSC curves in Figure 4b illustrate that the exothermic peak of PAN nanofibers, regardless of MXene content and incorporation method, occurs at approximately the same temperature (∼275°C), corresponding to the cyclization reactions of the nitrile groups in PAN in the absence of oxygen [42]. The consistency of this exothermic peak temperature across all nanofibers suggests that the stabilization process for the mats can be performed at the same or very similar temperatures. By increasing MXene loading in PAN nanofibers, the enthalpy (ΔH) decreased by 67%, from 612.5 J/g for the P mat to 202 J/g for the PM+M mat. This can be attributed to the presence of hydroxyl and carbonate surface functional groups on the MXene surface, supporting partial oxidation [43], thereby reducing the exothermicity of cyclization [44]. This reduction in enthalpy supports safer processing of carbon fibers during the stabilization stage, considering the risk of runaway exotherm at a larger scale. Notably, the P+M mat showed a slightly lower onset temperature, while the peak temperature did not decrease. This may indicate that while MXene coating slightly inhibits low‐temperature reactivity, it does not significantly affect the overall reaction behavior. Coating mats with MXene (468 J/g for P+M mat) has a slightly more pronounced effect on reducing the area of the exothermic peak compared to MXene incorporated into PAN (592 J/g for PM mat). The combination of MXene incorporation and coating (PM+M) resulted in the most significant peak reduction, suggesting that the heating time may need to be adjusted for the four mats to accommodate different reactivities of the materials. Table 1 summarizes key parameters obtained from the DSC curves, highlighting the effect of the different MXene treatments while confirming the presence of PAN conversion reactions in a typical temperature range.

**TABLE 1:** Key electrospun mats parameters obtained from DSC curves.

Precursor type	T_onset_ (°C)	T_peak_ (°C)	ΔH (J/g)
P	261.7	275.7	612.5
PM	261.7	276.7	592.4
P+M	254.5	277.3	468.1
PM+M	261.5	277.1	201.9

To estimate the expected yield of the material and verify the conversion process, thermogravimetric analysis (TGA) was performed on all four mats at different processing stages. The analysis was carried out under a nitrogen atmosphere for mats from all three stages of

processing: precursor, stabilized fibers, and carbonized material. The precursor mats, shown in Figure 4c, exhibited similar degradation behaviors, displaying a characteristic two‐step conversion process also highlighted by the peak temperatures from the first derivative of the degradation curves shown in Table 2 [45, 46]. The initial mass loss below 150°C corresponded to the removal of absorbed water and residual DMF from the Ti_3_C_2_T_x_ interlayers [47]. A significant mass loss observed around 280°C was indicative of the starting PAN polymer degradation. This highlights the critical role the stabilization process plays in converting PAN nanofibers into carbon nanofibers, as the PAN polymer transitions into a stabilized ladder‐like structure during this phase [36]. In addition to the effect of the MXene content, the comonomer composition of the PAN precursor, specifically the inclusion of methyl acrylate (MA) and itaconic acid (IA), plays a key role in determining the final carbon yield [42].

**TABLE 2:** Mass loss and first derivative (DTG) of the mass loss curves of the four tested materials.

	P	PM	P+M	PM+M
Remaining mass at 800°C (%)	37	41	48	48
Mass loss rate peak 1 (°C)	291	291	289	289
Mass loss rate peak 2 (°C)	421	414	412	412

The IA co‐monomer changes the cyclization reaction mechanism from free‐radical to ionic, facilitating higher chemical reactivity, leading to higher carbon yield [48]. The mass loading of Ti_3_C_2_T_x_ MXene in the fiber mats was estimated based on the residual mass of composite nanofiber mats at 1000°C in comparison with neat PAN nanofiber mats. A key observation is the increase in yield with higher MXene content, which differs depending on whether the MXene is integrated into the fibers or applied as a coating. Additionally, the mass loss occurs at slightly higher temperatures with increasing MXene content. The dip‐coating process results in a greater deposition of MXene on the mat surface, thereby enhancing the thermal stability of the composite films. All mats experience rapid mass loss between 270°C and 400°C. The yield for neat PAN is within the expected range (38% at 800°C), and as the MXene content increases, the final yield at 800°C rises by approximately 10%. Since MXene does not react or decompose within this temperature range, the overall yield for materials with higher MXene concentrations is correspondingly higher [49].

Figure 4d illustrates the thermal behavior of stabilized mats exposed to air for 1 h at 260°C. The decomposition began at 310°C, indicating that the fibers were effectively thermally stabilized during the stabilization process. The residual components of the stabilized mats (∼ %70) at 800°C were higher than those of the precursor mats (∼ 40%), clearly demonstrating that the stabilization procedure converts the material's microstructure and enables efficient conversion to carbon fibers during carbonization. A distinct difference is observed between MXene‐doped fibers, neat PAN, and MXene‐coated PAN. The MXene‐doped mat (PM) exhibited a lower yield, likely due to the MXene flakes inhibiting the stabilization reactions necessary for forming a uniform cyclized structure to some extent. The less‐developed structure resulted in an overall greater mass loss, as the material was not as thermally stable as neat PAN. The MXene‐coated mat (P+M) displayed the highest yield, combining a well‐cyclized structure with the MXene coating, which contributed to a higher overall mass retention at the end of the process.

Figure 4e presents the TGA thermographs of carbonized mats in a nitrogen atmosphere. For all carbonized mats, degradation improved. The PAN and PM+M mats showed higher yields, while the MXene‐incorporated (PM) and coated (P+M) mats exhibited lower yields, consistent with the results from earlier stages of processing. The TGA test was also performed in an air atmosphere, as shown in Figure 4f, where the yield differences became more pronounced compared to the test in a nitrogen atmosphere. The PAN mat demonstrated the lowest final yield, whereas the MXene‐coated carbonized mat retained nearly 40% of its initial mass at 1000°C. The results confirm that MXene integration techniques affect the performance of CNFs at high temperatures, which may be critical for specific applications.

These results further show that the thermal resistance of the carbonized mats is higher in a nitrogen atmosphere (800°C) than in air (400°C). Thermal degradation of the PM mat in air occurred at a higher temperature, approximately 450°C. The thermal degradation is linked to thermal conductivity, which can be increased by improving the orientation of basal planes and reducing pores, grains, and in‐plane lattice defects in the graphitic structures [50]. Given the increased flexibility of this type of mat (PM), it is well‐suited for high‐temperature applications in diverse environmental conditions.

### 2.2 Electrochemical Performance

**FIGURE 5:**
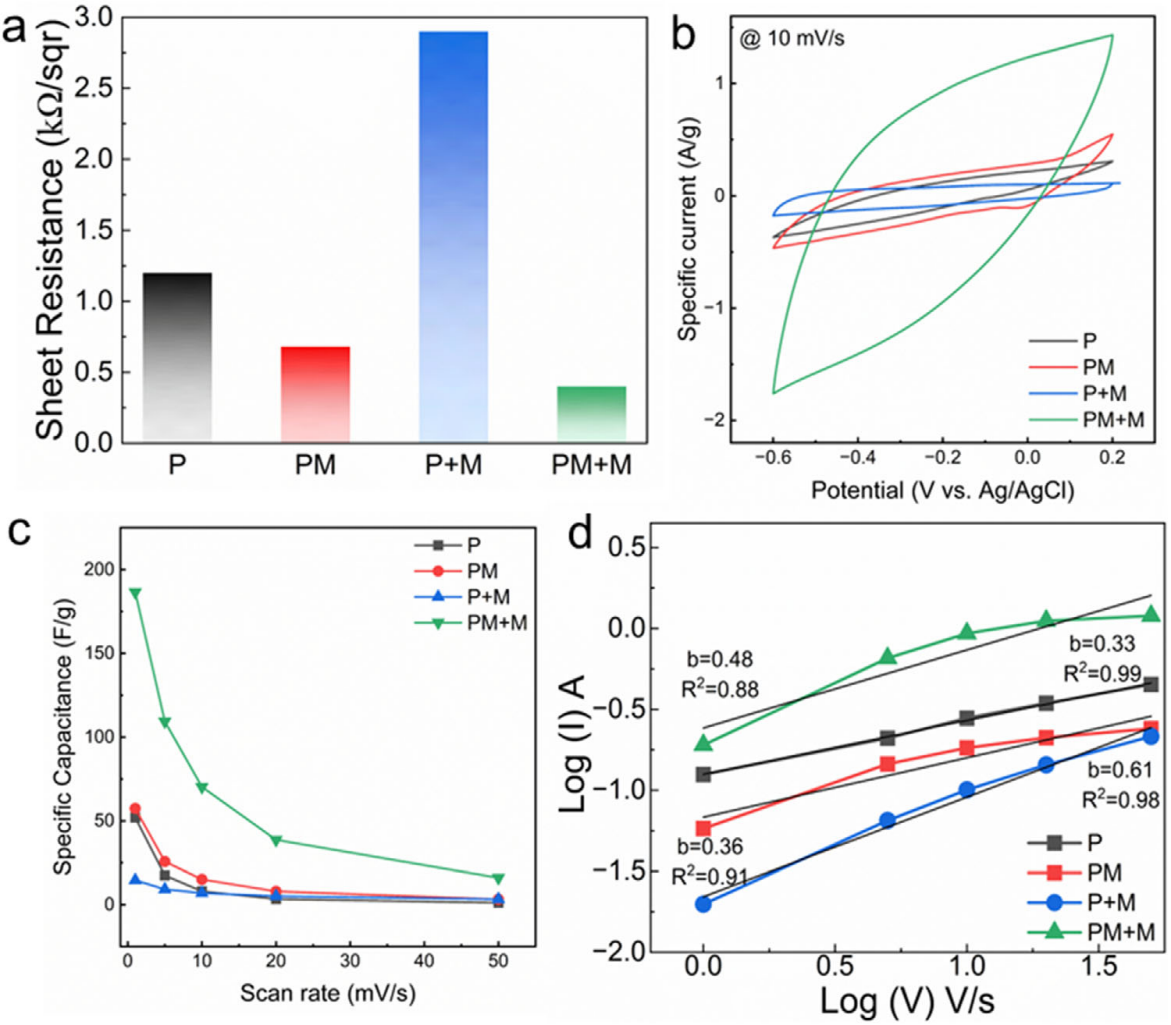
(a) comparison of sheet resistances of CNF mats, Electrochemical performance of carbonized nanofibers: (b) comparison of Cyclic voltammetry curves of all carbonized nanofibers at scan rate of 10 mV/s, (c) CV curves at various scan rates, (c) Capacitance trend of carbonized electrodes as a function of scan rate, (d) log(i) versus log(v) and calculation of b‐values.

Electrical conductivity is a critical parameter that influences the performance of electrodes in supercapacitors, helping to achieve high energy density by promoting electron and ion transfer during charge and discharge processes [51]. After carbonization, the sheet resistance (R_s_) of mats containing MXene both within and on the surface of the nanofibers (PM+M) was significantly lower (less than 0.5 kΩ/sqr). This is likely due to the continuous connection between MXene flakes inside and on the surface of the nanofibers [52], as shown in Figure 5a. The substantial difference in conductivity between carbonized PM and P+M mats can be attributed to the insertion of oxygenated functional groups, which disrupt the aromatic ladder‐like structure of the PAN in the P+M mat, which impedes the movement of electrons. The increased number of defects and disconnections within the carbon fiber mat reduces its conductivity [53]. During the stabilization process, micro and mesopores are generated in the PAN nanofibers, with the pore volume increasing as the thermal treatment time is extended. Embedding MXene flakes within PAN in the PM mat helps eliminate these pores, reduces the pore volume, thereby improving inter‐fiber connections that enhance conductivity [54]. Although the carbonization process results in a higher mass loss in the PM mat, the incorporation of MXene flakes within the nanofiber structure helps establish connections within the PAN ladder‐like configuration, improving its overall conductivity. Carbonization parameters (temperature and residence time) must be carefully controlled to prevent the formation of bulk titanium carbide (TiC) and titanium dioxide (TiO_2_), while maintaining high electrical conductivity. The oxidation of Ti_3_C_2_T_x_ MXene into TiO_2_ at elevated temperatures can have mixed effects on its electrochemical performance. While the formation of TiO_2_ can improve certain aspects like surface area and the creation of more active sites, it can also lead to a decrease in overall electrochemical activity due to the insulating nature of TiO_2_ and its potential dissolution [55].

The electrochemical performance of the fabricated electrodes for potential application in supercapacitors was evaluated (Figure 5b–d). MXene is recognized for its excellent specific capacitance and stable electrochemical performance in sulfuric acid (H_2_SO_4_) electrolyte [56]. The integration of MXenes with carbon nanofibers resulted in a uniform, highly porous morphology, enhanced surface area, and the formation of an effective conductive network. The CNFs were cut and assembled into a symmetric supercapacitor setup. Consequently, cyclic voltammetry (CV) measurements were conducted in 1 m H_2_SO_4_ using Ag/AgCl as the reference electrode, with scan rates ranging from 1 to 50 mV/s. The CV curve displayed in Figure 5b shows that at 10 mV s^−1^, a stable potential window of 0.6 V was maintained, indicating that the device has excellent reversibility characteristics. The incorporation of MXene flakes improved the hydrophilicity of CNFs, thereby facilitating better electrolyte penetration at the electrode/electrolyte interface and increasing the specific capacitance [23].

Figure 5c illustrates the capacitance trends over scan rate ranges, with the results aligning closely with the CV data (Figure S2). The maximum specific capacitance of 187 F/g at 1 mV/s was achieved with CNFs containing MXene both inside the fibers and as a coating (PM+M). In contrast, the capacitance of neat PAN carbon nanofibers under the same conditions reached 52 mF/cm^2^ at 1 mV/s. To investigate both faradaic and non‐faradaic charge storage, the power‐law equation *i* = *av^b^* can be used in combination with the current response of the CV curve, where *v* is the scan rate, *I* is the current, and a and b are constant parameters that can be determined by plotting log*v* vs log *i*. The b parameter varied between 0 to 1 to determine the nature of the charge storage. Generally, for b ≤ 0.5 indicates that the electrode material undergoes an ideal diffusion‐dominated faradic process [36]. The lower capacitance of P+M in comparison with the P mat is due to its higher resistance (Figure 5a).

Figure 5d presents the linear fit of the log(*i*) vs log(*ν*) plot at the redox peak potentials. The linear fit yields b values of 0.36 and 0.48 for PM+M and PM electrodes, respectively, which reflect ion diffusion driven by the porous structure of the electrodes. This, in turn, leads to a completely diffusion‐controlled process. These low b values at high scan rates are attributed to the mass diffusion‐limited reactions, which are thought to be caused by the relatively low active spots or the diffusion path in the electrode. In contrast, the MXene‐coated CNFs (P+M) exhibited a b value of 0.61 (where 0.5 ≤ b ≤ 1), suggesting that the ion storage dynamics in the P+M electrode exhibit pseudocapacitive characteristics [19]. The higher value for b signifies the improvement of surface accessibility and ion diffusion in the electrodes, resulting in greater rate performance and capacitance retention [56].

## 3 Conclusion

To clearly understand the effect of MXene integration methods on the thermal and electrochemical behavior of CNFs, it is essential to keep fabrication and process parameters consistent. Maintaining consistent variables such as electrospinning conditions, MXene flake size and concentration, PAN precursor composition, and carbonization temperature and duration is essential for ensuring accurate comparisons. This consistency allows for a more reliable evaluation of different MXene integration approaches and helps identify the most suitable method for a specific application.

This study offers insights into how various MXene incorporation strategies, along with consistent process parameters, influence the thermal and electrochemical performance of the resulting CNF mats. During thermal stabilization, neat PAN mats exhibited the highest shrinkage and lowest yield. Incorporating MXene into the electrospinning solution resulted in more flexible CNF mats. The synergy between MXene incorporation and coating resulted in a reduction in exothermic enthalpy during stabilization, facilitating safer carbon fiber processing. Increased MXene content in the nanofibers led to the highest conductivity, demonstrating significant potential for enhanced energy storage performance. However, in this method, the restacking of MXene flakes impeded ion diffusion within the electrode matrix, limiting charge delivery rates. While the incorporation of MXene into the nanofiber solution improves energy storage capacity, dip coating enhances ion transport, enabling faster charge/discharge cycles. MXene‐functionalized CNFs exhibit high thermal stability and enhanced electrochemical properties, positioning them as promising candidates for electrodes in advanced batteries as well as highly porous, conductive mats for high‐temperature and harsh‐environment applications. In our future work we will explore strategies to mitigate MXene restacking, such as interlayer spacers, surface functionalization and hydrothermal treatment to further enhance ion accessibility and charge transport. Additionally, long‐term cycling stability performance under realistic operating conditions will be investigated to better assess the practical viability of these materials in real energy storage systems.

## 4 Experimental Section

### 4.1 Materials

The PAN precursor used was commercial carbon fiber grade PAN fiber containing itaconic acid and methyl methacrylate. The Ti_3_AlC_2_ precursor was provided by Carbon‐Ukraine Ltd. Lithium fluoride (LiF, 99% purity), hydrochloric acid (HCl, 12 M), acetone, and N,N‐dimethylformamide (DMF, ≥99%) were sourced from Sigma–Aldrich Pty Ltd. MXenes were synthesized from the MAX phase precursor using the MILD etching process, as described in earlier work [30].

### 4.2 Electrospinning and Dip‐Coating

After removing the spin finish on the PAN precursor fibers with hot water and acetone, PAN was then dissolved in N,N‐dimethylformamide (DMF) and stirred continuously with a magnetic stirrer for 12 h at 60°C until fully dissolved. After the synthesis process, MXene flakes dispersed in water were redispersed in dimethylformamide (DMF) using a solvent exchange (SE) process for incorporation into the PAN solution used in electrospinning. In this process, the aqueous MXene dispersion was centrifuged at 15 000 rpm (24 630 × g) for one hour. The supernatant was discarded and replaced with an equal volume of DMF, followed by vigorous shaking. This procedure was repeated three times to ensure complete replacement of water with DMF. MXene sheets exhibit good stability in DMF. The redispersed MXene was subsequently sonicated using a probe sonicator (QSonica Q700) at 50% amplitude and 52 W power, with a pulse‐on time of 2 min and a pulse‐off time of 5 s, for a total duration of 30 min [24]. Sonication reduced the MXene sheet size to the nanoscale (hundreds of nanometers), which promotes uniform dispersion in the PAN solution. MXene was then added to the electrospinning solution, which now contained 12 wt.% PAN and 1 wt.% MXene. The solution was then transferred to a 10 mL syringe, connected to an 18‐gauge metal needle. Consistent electrospinning conditions were maintained for all fabricated mats, using a voltage of 20 kV, a tip‐to‐collector distance of 20 cm, and a feed flow rate of 0.8 mL/h. A grounded rotating drum (100 rpm) was used as the fiber collector. By controlling the spinning time, the thickness of the resulting PAN nanofibrous mats was carefully adjusted. For the dip‐coated mats, the material was immersed in a mixture of MXene (10 mg/mL) in ethanol/water (70/30) and subsequently dried in an oven at 60°C overnight.

### 4.3 Stabilization and Carbonization of Electrospun Mats

Following electrospinning, the mats were gently removed from the aluminum foil and stabilized in ambient air using a fan‐forced laboratory oven (Thermo Scientific). The oven was heated to a temperature of 260°C, using a heating rate of 2°C/min, and held at times between 30 and 60 min to determine the optimal period for the desired degree of stabilization. Carbonization of the electrospun mats was subsequently performed using a three‐zone pilot‐scale furnace from a carbon fiber line at Carbon Nexus, Deakin University. The furnace temperatures in each zone were set to 400°C, 600°C, and 900°C, respectively, with an inert nitrogen (N_2_) atmosphere. The nanofibers passed through the furnace at a speed of 10 m/h, with a total residence time of 9 min equally divided between the three zones.

### 4.4 Characterization

The heat flow and overall heat of reaction for the precursor material were determined using a DSC 214 Polyma differential scanning calorimeter (DSC) (Netzsch, Germany). Approximately 5 mg of fiber material was carefully weighed and sealed in an aluminum crucible. The sample was then placed into the furnace of the instrument, which was purged with 40 mL/min of nitrogen. The sample was heated to a minimum of 300°C at a rate of 5°C/min, with the results including peak temperatures and enthalpies analyzed using the accompanying Proteus software (Netzsch, Germany). The evolution of the nanofiber chemistry during the stabilization stage was measured using a Bruker Lumos FTIR spectrometer equipped with a germanium crystal. The EOR index was calculated using Equation 1. Where *Abs_(1590)_* and *Abs_(2242)_* were the measured absorbance of the C═C, C═N, and C≡N functional groups, respectively [57].

(1) $$EOR\left(\%\right)=\frac{(100\times 0.29\times Abs_{\left(1590\right)}}{(Abs_{\left(2242\right)}+(0.29\times Abs_{\left(1590\right))}}\;$$

Thermal degradation of the material was evaluated using a thermogravimetric analyzer (TGA), TG 209 F1 Libra (Netzsch, Germany). Approximately 5 mg of fiber material was weighed and placed into an aluminum oxide crucible. The sample was then introduced into the furnace chamber, which was purged with 20 mL/min of nitrogen or air, depending on the required temperature range for the analysis. The furnace was heated to 800°C at a rate of 5°C/min, and transition temperatures were determined using the Proteus software (Netzsch, Germany).

A 50N load‐cell‐equipped Instron universal testing machine was used to evaluate the mechanical properties of the electrospun mats. Pneumatic tensile grips ensured secure clamping, and a preload of 0.1 N was applied to remove any slack in the specimens. Subsequently, the samples were subjected to tensile testing at a strain rate of 1 mm/min.

Raman spectra of the carbon nanofiber mats were obtained using a Raman Micro Spectrometer (Renishaw plc, Gloucestershire, UK), equipped with a 514 nm argon laser. Electrochemical tests, including cyclic voltammetry (CV), electrochemical impedance spectroscopy (EIS), and electrochemical cycling tests, were performed using a VMP3 electrochemical workstation (BioLogic SP 300, France) in a three‐electrode setup. The electrolyte used was 1 m sulfuric acid (H_2_SO4), and the measurements were conducted at room temperature. In the three‐electrode setup, the carbon nanofibers served as the working electrodes, connected to a glassy carbon electrode holder (Gaossunion GC‐2). Ag/AgCl (3 m KCl with a salt bridge) was used as the reference electrode, and a graphite rod served as the counter electrode. The Ag/AgCl reference electrode was separated from the H_2_SO_4_ electrolyte by using a salt bridge.

## Conflicts of Interest

The authors declare no conflicts of interest.

## Supporting information




**Supporting File 1**: marc70033‐sup‐0001‐SuppMat.docx.

## Data Availability

The data that support the findings of this study are available from the corresponding author upon reasonable request.
