# Supplementary material for: Functionalizing PAN Carbon Nanofibers Using Ti3C2Tx MXene for Improved Thermal and Electrochemical Behavior
Source: Macromol Rapid Commun. 2025 Aug 20;46(21):e00429. doi: 10.1002/marc.202500429 (PMC12590925; doi:10.1002/marc.202500429)
Supplement: Supplementary file 1 — Supporting File 1: marc70033‐sup‐0001‐SuppMat.docx. [file MARC-46-e00429-s001.docx]

Supporting Information

Functionalizing PAN Carbon Nanofibers using Ti₃C₂Tₓ MXene for improved Thermal and Electrochemical Behavior

*Fatemeh Mokhtari^1*^, Thomas Groetsch^2^, Pejman Heidarian^2^, Maxime Maghe^2^, Russell J. Varley^3*^*

^1^ Department of Materials Engineering, KU Leuven, Leuven 3001, Belgium

^2^ Carbon Nexus at the Institute for Frontier Materials, Deakin University, Waurn Ponds, Victoria 3216, Australia

^3^ School of Engineering, RMIT University, Melbourne, VIC 3001, Australia

E-mail: [Fatemeh.mokhtari@kuleuven.be](mailto:Fatemeh.mokhtari@kuleuven.be), Russell.varley2@rmit.edu.au

**Supporting figures:**


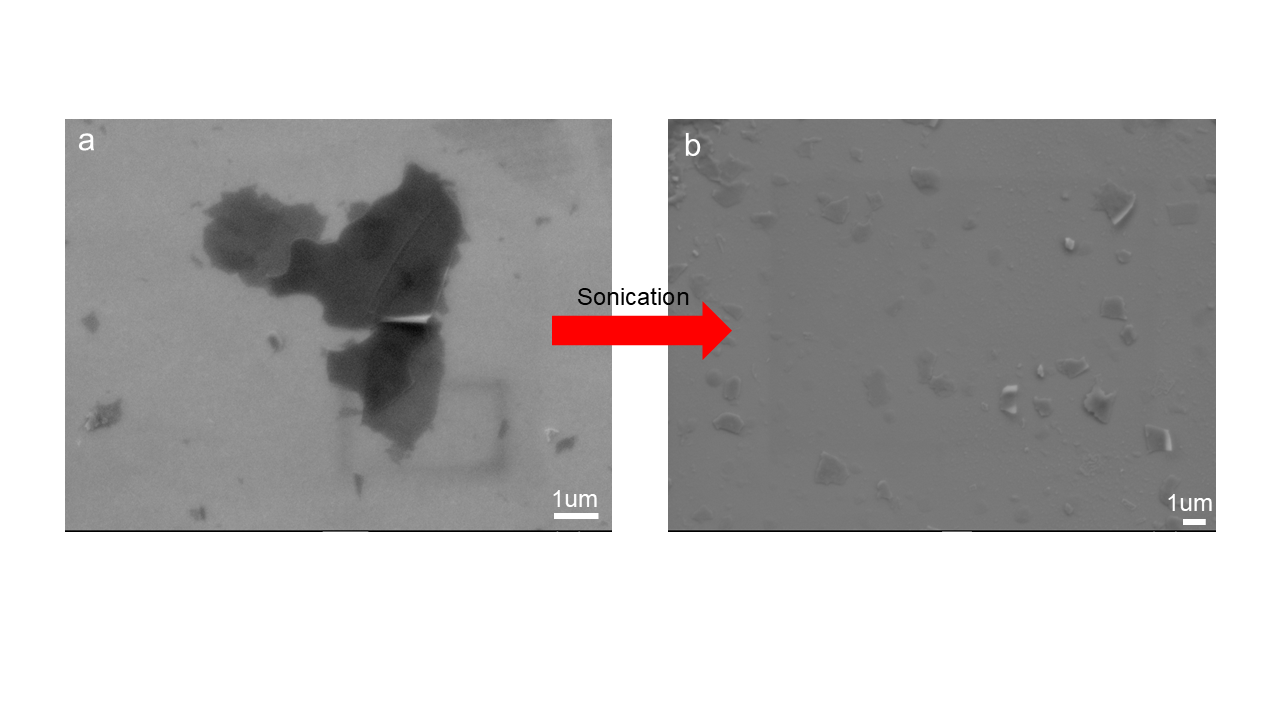


**Figure S1**. SEM images of : a) The synthesized Ti₃C₂Tₓ MXene flakes, b) MXene flakes after solvent exchange and prob sonication.


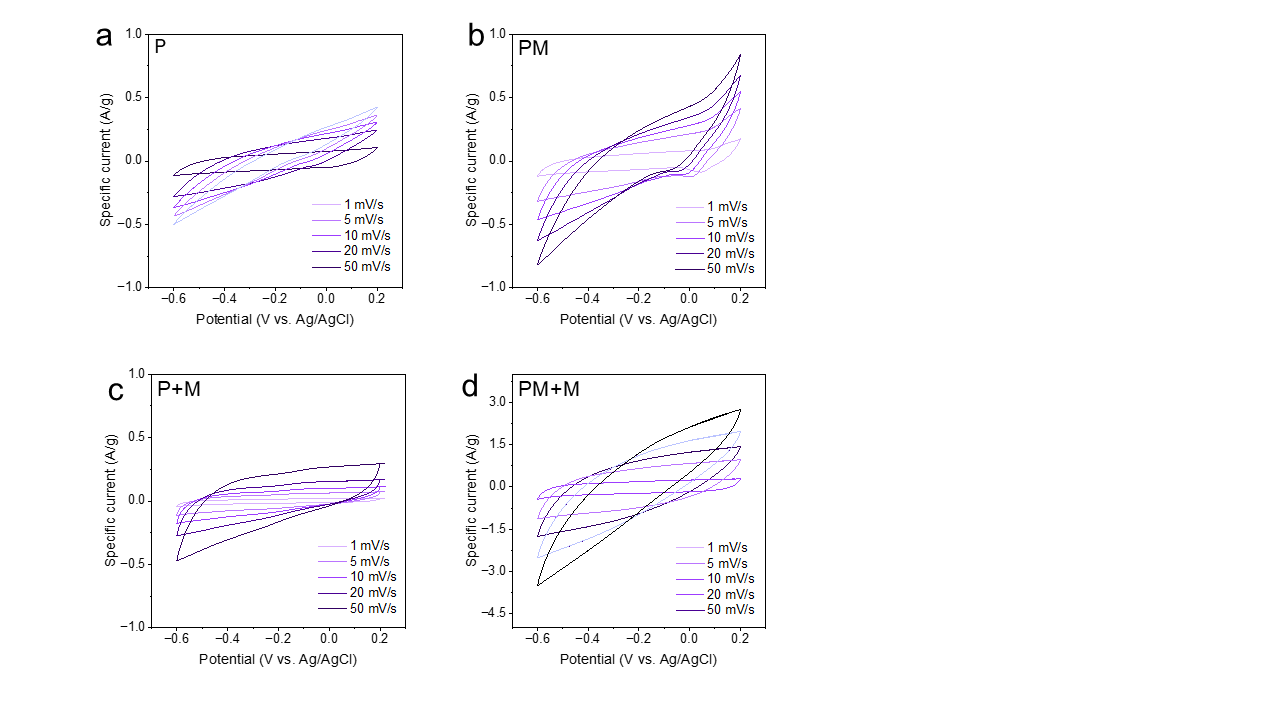


**Figure S2**. CV curve of CNFs at different scan rates: a) P, b) PM, c) P+M, and d) PM+M
